# Exploring the Prevalence of Ten Polyomaviruses and Two Herpes Viruses in Breast Cancer

**DOI:** 10.1371/journal.pone.0039842

**Published:** 2012-08-15

**Authors:** Annika Antonsson, Seweryn Bialasiewicz, Rebecca J. Rockett, Kevin Jacob, Ian C. Bennett, Theo P. Sloots

**Affiliations:** 1 Queensland Institute of Medical Research, Department of Population Health, Herston, Brisbane, Queensland, Australia; 2 Queensland Paediatric Infectious Diseases Laboratory, Queensland Children's Medical Research Institute, Sir Albert Sakzewski Virus Research Centre, Queensland Children's Health Service and The University of Queensland, Brisbane, Queensland, Australia; 3 Department of Surgery, Breast & Endocrine Unit, Princess Alexandra Hospital, Woolloongabba, Brisbane, Queensland, Australia; National Cancer Institute, United States of America

## Abstract

Several different viruses have been proposed to play a role in breast carcinogenesis. The aim of this study was to investigate the prevalence of a subset of viruses in breast cancer tissue. We investigated the prevalence of 12 DNA viruses: EBV and CMV from the *Herpesviridae* family and SV40, BKV, JCV, MCV, WUV, KIV, LPV, HPyV6, HPyV7, and TSV from the *Polyomaviridae* family in 54 fresh frozen breast tumour specimens. Relevant clinical data and basic lifestyle data were available for all patients. The tissue samples were DNA extracted and real-time PCR assays were used for viral detection.

The highest prevalence, 10% (5/54), was found for EBV. MCV, HPyV6, and HPyV7 were detected in single patient samples (2% each), while WUV, KIV, JCV, BKV, LPV, SV40, TSV and CMV were not detected in the 54 breast cancer specimens analysed here. Further investigations are needed to elucidate the potential role of viruses, and particularly EBV, in breast carcinogenesis.

## Introduction

Breast cancer is the most common type of cancer in women worldwide [1]. Only a small proportion of breast cancer cases, approximately 5%, are caused by hereditary mutations (such as BRCA1 and 2) [2], with the majority of breast cancers being sporadic or acquired in nature. Whilst there are some recognised risk factors for sporadic breast cancer such as late first full-term pregnancy, nulliparity, obesity and alcohol, only a small proportion of breast cancer cases actually display these risk factors. Hence, the aetiology of sporadic breast cancer remains enigmatic. Infectious agents have been estimated to be responsible for 18% of human cancers [3], and a role of viral infection in breast carcinogenesis has been proposed. A number of studies have examined the role of different viruses with oncogenic potential such as human papillomavirus (HPV), mouse mammary tumour virus (MMTV), simian virus 40 (SV40), cytomegalovirus (CMV) and Epstein-Bar virus (EBV) in breast cancer tissue [4], [5], [6]. However, even though these viruses have been identified in varying prevalence in breast cancer tissue, there has so far not been any definitive evidence for a causal role of any of these viruses [4], [7].

SV40 is a non-human member of the *Polyomaviridae* family which has traditionally contained only two true human-host specific viruses (JCV and BKV). In the last four years, there has been a flurry of new human polyomaviruses discovered, however their role in various human cancers has not been thoroughly evaluated. In 2007, WU and KI polyomaviruses (WUV and KIV, respectively) were nearly simultaneously discovered by separate groups in the respiratory tracts of children presenting with respiratory disease [8], [9]. A year later, Merkel Cell Polyomavirus (MCV) was discovered integrated into Merkel cell carcinoma (MCC) tissue, a rare but aggressive form of skin cancer [10]. Subsequent studies have confirmed the association between MCV, viral integration and MCC (up to 80% tumour positivity rate), and to this day, MCV presents the strongest evidence for polyomavirus involvement in human oncogenesis [11], [12]. More recently, three additional polyomaviruses have been discovered; HPyV6 and HPyV7 on the skin of healthy human volunteers [13] and Trichodysplasia Spinulosa Polyomavirus (TSV) from the hair follicles of a heart transplant patient suffering from TSV's namesake disease [14]. Like SV40, Lymphotropic Polyomavirus (LPV) is a monkey-origin virus [15], however in the last few years, there have been several reports of its detection in the blood of immunocompromised patients [16], [17]. The new members of the human polyomavirus family appear to have a tissue tropism preference for various forms of epithelial tissues, which also form the majority of breast cancer types [18].

In this study we investigated the prevalence of two members of the *Herpesviridae* family (EBV and CMV) together with ten members of the *Polyomaviridae* family (SV40, BKV, JCV, MCV, WUV, KIV, LPV, HPyV6, HPyV7 and TSV) in breast tumour specimen.

## Materials and Methods

### Study population and samples

We analysed 54 breast cancer tissue samples that were removed as a part of treatment from patients undergoing surgery at the Wesley Hospital, Brisbane, Queensland, Australia between 2003 and 2007. These samples were used in a previously published study into the role of human papillomaviruses in breast cancer [6]. All tissue samples were confirmed by histology and the patients' median age was 57 years, with a range of 31–88 years. We also had adjacent tumour free breast tissue from 10 of these women that served as controls. The median age for these 10 women was 53. Data on diagnosis, location, invasive grade, histological type, menopausal status, family history of breast cancer, cervical cancer diagnosis and HER2, oestrogen and progesterone receptors was also captured in an Access database.

Written informed consent was obtained from all patients, and the taking of fresh frozen breast cancer tissue samples for this project was approved by the Princess Alexandra Hospital Human Research Ethics Committee (PAH 2007/057).

Tumour tissue samples were snap frozen and stored at −80°C. Prior to extraction the samples were grinded in 1.5 ml tubes on dry ice. The tissue samples were then homogenized in Trizol (Invitrogen, Carlsbad, CA), and DNA extraction was carried out following the protocol provided by the manufacturer. The DNA extracted from the breast tissue specimens was stored at −20°C until analysed.

### Viral analysis

Viral detection was achieved with both published and specifically designed real-time PCR (rtPCR) assays. Samples were screened in duplex for JCV (JL1) and BKV (V3a) using previously published methods [19], [20]. Similarly, polyomaviruses TSV (VP1 assay) [14], SV40 (SL1 assay) [20], as well as EBV [21], and CMV [22] were screened for in previously published singleplex assays. Based on available genomic sequences, rtPCR assays targeting MCV, LPV, HPyV6, HPyV7, WUV and KIV were designed and evaluated for cross-reaction with other viral species (Table 1). The LPV, HPyV6 and HPyV7 assays were run as singleplex reactions, while the WUV and KIV assays were combined into a triplex.

**Table 1:** Primer and probe sequences, in 5′ to 3′ orientation, of LPV, HPyV6, HPyV7, WUV and KIV assays used in this study, along with their genomic target.

Oligo	Sequence	Virus	Target
LPV-VP2-F	CATTGAAATAGAAGCAGTGGATCTTG	LPV	VP2
LPV-VP2-R	AAACTCCTATTCCTATGGCATTGTTG		
LPV-VP2-Prb	AGAGCAGTTTTCCCTCCTAAGTGCTATCCCAA		
HPyV6-VP2-F	TTGAGGAGCTGGACAAAGAGATT	HPyV6	VP2
HPyV6-VP2-R	TCTGGGAAGCTTTTGAATTGGT		
HPyV6-VP2-Prb	AGGAAGATGCCTTGTCACAGAAAAGGAAATG		
HPyV6-LT-F	ACCAGGTGGGTGATGAAGACA	HPyV6	LTAg
HPyV6-LT-R	CGCCTGAATGTTTTAAAGGAGAA		
HPyV6-LT-Prb	TTGGTCCCTCAGGGTGGCATTCA		
HPyV7-LT-F	AAGACATTCAGTCTTTGCATTTTCTG	HPyV7	LTAg
HPyV7-LT-R	CCCCTCATACAGCATAAGGTTAGATT		
HPyV7-LT-Prb	CCACCTTTATCTGGATGATACTTTTTGCTGGC		
HPyV7-VP2-F	GAGGAAGGAAACACTCCCCAGTA	HPyV7	VP2
HPyV7-VP2-R	TTCACTTCTTTTTGTAGCTCCTCAAG		
HPyV7-VP2-Prb	ACTATACCTCAATGGATGCTTTTTGT		
WUV-F-Reg-F	GCCGACAGCCGTTGGATATA	WUV	NCCR
WUV-F-Reg-R	TTTCAGGCACAGCAAGCAAT		
WUV-F-Reg-Prb	AGGGTCACCATTTTTATTTCAGATGGGCA		
KIV-D-LT-F	CACAGGTGGTTTTCTATAAATTTTGTACTT	KIV	LTAg
KIV-D-LT-R	GAATGCATACATCCCACTGCTTC		
KIV-D-LT-Prb	TGCATTGGCATTCGTGATTGTAGCCA		
KIV-E-Reg-F	GAACTTCTACTGTCCTTGACACAGGTA	KIV	NCCR
KIV-E-Reg-R	GGATTAGAACTTACAGTCTTAGCATTTCAG		
KIV-E-Reg-Prb	TGGGAAACATCCGGTTTCCTCTCACTTCC		
MCV-LT1.1-F	AGCTCAGAAGTGACTTCTCTATGTTTGA	MCV	LTAg
MCV-LT1.1-R	ACAATGCTGGCGAGACAACT		
MCV-LT1.1-Prb	TTTGCAGAGGTCCTGGGTGCATG		

LTAg = Large T Antigen, NCCR = Non Coding Control Region.

Samples positive by the HPyV6-VP2 and HPyV7-LT screening assays were confirmed with the use of secondary rtPCR assays HPyV6-LT and HPyV7-VP2 (Table 1), respectively, while MCV-LT1.1 positive samples were confirmed by a previously described MCV rtPCR assay [23].

A common reaction mix was used for all rtPCR assays. Briefly, the rtPCR consisting of 12.5 µl Quantitect Probe PCR Mix (QIAGEN, Australia), 10 pmol of each primer, 4 pmol of each probe and 2 µl of template in a 25 µl final reaction was performed in a Rotorgene 3000 (Corbett Research, Sydney, Australia) under the following conditions: 15 minutes incubation at 95°C, followed by 45 cycles of 95°C for 15 seconds and 60°C for 1 minute.

For positive controls, the MCV, WUV/KIV, JCV/BKV, CMV, and EBV screening assays used known clinical positive samples, for the LPV and TSV screening assays whole-genome plasmids were used, for the HPyV6 and HPyV7 screening assays synthetic oligo controls were used, and for the SV40 screening assay the SV40-integrated COS-1 cell line was used.

Only samples which were both positive for the screening and confirmatory assays were considered “positive”. Other sample targets which were well characterised, such as JCV, BKV, EBV, CMV, WUV, KIV, or did not produce any positives (LPV, TSV, SV40) did not need confirmation.

A PCR assay targeting the human L1 sequence was used to ensure that our sample contained human DNA and that no PCR inhibiting agents were present, as described in our previous study [6].

### Statistical analysis

For comparisons, we used the t-test for normally distributed continuous variables and chi-square or Fisher's test (for small samples where the expected number in any cell was <5) for categorical variables. All analyses were conducted in SAS (version 9.2) and all significance tests were two sided at α = 0.05.

## Results and Discussion

Fifty-four Australian breast cancer tumours were analysed for the presence of ten different viruses from the herpes and polyomavirus families.

The mean age of the 54 women studied was 57 years and the mean size of the study tumours was 24 mm (range: 8–120 mm), with a majority of patients diagnosed with invasive ductal carcinoma (87%). Other relevant clinical features for the 54 patients studied can be found in Table 2.

**Table 2:** Basic characteristic for study participants.

		N	%
Total		54	
Age	(mean and SD)	57.0 (11.9)	
Menopausal status	Pre	13	24%
	Peri	9	17%
	Post	32	59%
Tumour size	(mean and SD)	24.3 (23.0)	
Tumour size	>25 mm	41	76%
	<25 mm	13	24%
Cancer type	Invasive Ductal	47	87%
	mixed	1	2%
	DCIS	1	2%
	Invasive Lobular	5	9%
Grade	1	10	19%
	2	23	42%
	3	20	37%
	N/A	1	2%
Nodal Status	Positive	20	37%
	Negative	34	63%
ER	Positive	43	80%
	Negative	11	20%
PR	Positive	39	72%
	Negative	15	28%
HER2	Positive	8	15%
	Negative	46	85%
Triple negative	Yes	6	11%
(ER, PR and HER2)	No	48	89%

The highest prevalence of the viruses investigated here in the breast tumour samples was 10% (5/54) which was found for EBV. None of the ten adjacent healthy tissues were EBV positive, including one originating from an EBV positive patient. Four of five EBV positive samples were grade 3 tumours, with the remaining one being grade 2. All five EBV positive women were postmenopausal, and were on average 10 years older than the EBV negative women (66 and 56 years old, respectively), however this age difference was not statistically significant (p = 0.09). In a recent paper, the EBV positive cells in breast tumours were found to be infiltrating lymphocytes, while no virus was detected in the malignant breast cells [24]. Due to insufficient original sample quantities, we were not able to perform *in situ* hybridisation or immunohistochemistry on the EBV positive samples, and thus, could not determine the cellular origin of the detected virus.

MCV, HPyV6, and HPyV7 were detected in single cases (2% each) of the breast tumour samples. The MCV positive patient was a 58 year old woman with a grade 1 tumour of mixed type (ductal and lobular) breast cancer. The patient with the HPyV6 positive tumour was 52 years and had a grade 2 ductal breast tumour, while the HPyV7 positive patient was 78 years and had a grade 2 lobular breast tumour. The MCV, HPyV6 and HPyV7 positive samples produced high cycle threshold values of 31.9, 38.5, and 40.2, respectively, which is suggestive of low viral genome copy numbers. Given the evident low viral loads, and that MCV, HPyV6 and HPyV7 are polyomavirus with skin tropism, it is unclear whether these findings represent true infection or merely sample contamination originating from the skin during surgical excision or from skin shedding during sample processing.

WUV, KIV, JCV, BKV, LPV, SV40, TSV, and CMV were not detected in the 54 breast cancer specimens analysed in this study.

JCV and BKV were discovered 40 years ago and findings of these two viruses have been reported in various types of tissues, but infections have mainly been associated with urinary tract diseases and mild upper respiratory tract infections [25]. Both viruses have been found to persist in kidneys, B-lymphocytes and the central nervous system (CNS) [26], [27], [28]. There are, to our knowledge, only one publication to date investigating JCV and BKV DNA in breast tumours [29]. This recent paper by Hachana *et al* performed on Tunisian breast tumours did not find any BKV DNA positive tumours, but 23% of the specimens were found to be JCV positive. The JCV positive tumours in this paper were all invasive ductal type carcinomas. The majority of our samples (87%) were also of invasive ductal type, but we did not identify JCV in our population. Real-time PCR is generally accepted to be at least one log more sensitive than conventional PCR, and increasing PCR cycle numbers tends to increase the final yield and sensitivity of the assay. Considering Hachana *et al* used 35 cycles on their conventional PCR for the detection of JCV, while we used 45 cycles in a real-time PCR assay, we believe that assay sensitivity cannot explain the divergent JCV detections. In view of the Tunisian samples and our samples being similar breast tumour type (majority invasive ductal), and the previous study's apparent lower JCV assay sensitivity, it is difficult to explain the vast difference in JCV detections between our sample sets. Furthermore, Hachana *et al* found a significant correlation between multiple viral infection (SV40, JCV and MMTV) and “triple negative” phenotype (ER, PR and HER2) [30]. Due to the low number of polyomavirus detections, we did not find any correlations with the “triple negative” phenotype and any other variables in our dataset.

None of our specimens were positive for the monkey origin polyomavirus SV40. A study by Hachana *et al.* [30] investigating SV40 in breast tumours found 22% of tumours to be SV40 positive compared to 2% in matched tumour-free breast tissue, which contrasts with the findings of our study. The previous study SV40 assay targeted the large T antigen gene, whereas we used a previously published assay targeting the structural VP2 gene. This choice of targets may have impacted our ability to detect any integrated SV40 genomes if their structural genes were truncated. Alternatively, the detection of SV40 DNA may have been due to endogenous contamination of reagents or samples by SV40 sequences, considering the ubiquity of SV40 sequence use in molecular biology.

CMV is an interesting virus in regard to breast cancer, even though there is no solid evidence of an involvement in breast carcinogenesis. Thirty to 40% of CMV infections are acquired during the first year of life and the route of transmission is mainly through breast milk from the mother [31]. Interestingly, one case-control study found an odds ratio (OR) of 4.0 for women seroconverting to CMV at least four years prior to being diagnosed with breast cancer compared to women who did not seroconvert [32]. When adjusting for parity and age at first birth, the OR for CMV seroconversion increased to 9.7 in the same study. However, CMV was not detected in our study population.

To conclude, we found very low or no prevalence to the viruses investigated in this study apart from EBV, although due to the histological limitations of the study, we could not draw robust conclusions on EBV's role in breast cancer. The study data suggests the lack of these DNA viruses' involvement in breast cancer, however further investigations and larger studies are needed to elucidate if EBV plays a role in breast carcinogenesis.

## References

[1] Ferlay J, Shin HR, Bray F, Forman D, Mathers C, et al (2010) Estimates of worldwide burden of cancer in 2008: GLOBOCAN 2008. Int J Cancer 127: 2893–2917. 21351269 10.1002/ijc.25516

[2] Campeau PM, Foulkes WD, Tischkowitz MD (2008) Hereditary breast cancer: new genetic developments, new therapeutic avenues. Hum Genet 124: 31–42. 18575892 10.1007/s00439-008-0529-1

[3] Parkin DM (2006) The global health burden of infection-associated cancers in the year 2002. Int J Cancer 118: 3030–3044. 16404738 10.1002/ijc.21731

[4] Amarante MK, Watanabe MA (2009) The possible involvement of virus in breast cancer. J Cancer Res Clin Oncol 135: 329–337. 19009309 10.1007/s00432-008-0511-2 PMC12160138

[5] Lawson JS, Heng B (2010) Viruses and Breast Cancer. Cancers 2: 752–772. 24281093 10.3390/cancers2020752 PMC3835103

[6] Antonsson A, Spurr TP, Chen AC, Francis GD, McMillan NA, et al (2011) High prevalence of human papillomaviruses in fresh frozen breast cancer samples. J Med Virol 83: 2157–2163. 22012724 10.1002/jmv.22223

[7] Lawson JS, Gunzburg WH, Whitaker NJ (2006) Viruses and human breast cancer. Future Microbiol 1: 33–51. 17661684 10.2217/17460913.1.1.33

[8] Allander T, Andreasson K, Gupta S, Bjerkner A, Bogdanovic G, et al (2007) Identification of a third human polyomavirus. J Virol 81: 4130–4136. 17287263 10.1128/JVI.00028-07 PMC1866148

[9] Gaynor AM, Nissen MD, Whiley DM, Mackay IM, Lambert SB, et al (2007) Identification of a novel polyomavirus from patients with acute respiratory tract infections. PLoS Pathog 3: e64. 17480120 10.1371/journal.ppat.0030064 PMC1864993

[10] Feng H, Shuda M, Chang Y, Moore PS (2008) Clonal integration of a polyomavirus in human Merkel cell carcinoma. Science 319: 1096–1100. 18202256 10.1126/science.1152586 PMC2740911

[11] Foulongne V, Dereure O, Kluger N, Moles JP, Guillot B, et al (2010) Merkel cell polyomavirus DNA detection in lesional and nonlesional skin from patients with Merkel cell carcinoma or other skin diseases. Br J Dermatol 162: 59–63. 19678822 10.1111/j.1365-2133.2009.09381.x

[12] Sastre-Garau X, Peter M, Avril MF, Laude H, Couturier J, et al (2009) Merkel cell carcinoma of the skin: pathological and molecular evidence for a causative role of MCV in oncogenesis. J Pathol 218: 48–56. 19291712 10.1002/path.2532

[13] Schowalter RM, Pastrana DV, Pumphrey KA, Moyer AL, Buck CB (2010) Merkel cell polyomavirus and two previously unknown polyomaviruses are chronically shed from human skin. Cell Host Microbe 7: 509–515. 20542254 10.1016/j.chom.2010.05.006 PMC2919322

[14] van der Meijden E, Janssens RW, Lauber C, Bouwes Bavinck JN, Gorbalenya AE, et al (2010) Discovery of a new human polyomavirus associated with trichodysplasia spinulosa in an immunocompromized patient. PLoS Pathog 6: e1001024. 20686659 10.1371/journal.ppat.1001024 PMC2912394

[15] zur Hausen H, Gissmann L (1979) Lymphotropic papovaviruses isolated from African green monkey and human cells. Med Microbiol Immunol 167: 137–153. 226854 10.1007/BF02121180

[16] Delbue S, Tremolada S, Branchetti E, Elia F, Gualco E, et al (2008) First identification and molecular characterization of lymphotropic polyomavirus in peripheral blood from patients with leukoencephalopathies. J Clin Microbiol 46: 2461–2462. 18480226 10.1128/JCM.00381-08 PMC2446883

[17] Delbue S, Tremolada S, Elia F, Carloni C, Amico S, et al (2010) Lymphotropic polyomavirus is detected in peripheral blood from immunocompromised and healthy subjects. J Clin Virol 47: 156–160. 20042367 10.1016/j.jcv.2009.11.029 PMC2818589

[18] Weigelt B, Reis-Filho JS (2009) Histological and molecular types of breast cancer: is there a unifying taxonomy? Nat Rev Clin Oncol 6: 718–730. 19942925 10.1038/nrclinonc.2009.166

[19] Hirsch HH, Mohaupt M, Klimkait T (2001) Prospective monitoring of BK virus load after discontinuing sirolimus treatment in a renal transplant patient with BK virus nephropathy. J Infect Dis 184: 1494–1495; author reply 1495–1496. 11709797 10.1086/324425

[20] Pal A, Sirota L, Maudru T, Peden K, Lewis AM Jr (2006) Real-time, quantitative PCR assays for the detection of virus-specific DNA in samples with mixed populations of polyomaviruses. J Virol Methods 135: 32–42. 16527364 10.1016/j.jviromet.2006.01.018

[21] Kimura H, Morita M, Yabuta Y, Kuzushima K, Kato K, et al (1999) Quantitative analysis of Epstein-Barr virus load by using a real-time PCR assay. J Clin Microbiol 37: 132–136. 9854077 10.1128/jcm.37.1.132-136.1999 PMC84187

[22] Watzinger F, Suda M, Preuner S, Baumgartinger R, Ebner K, et al (2004) Real-time quantitative PCR assays for detection and monitoring of pathogenic human viruses in immunosuppressed pediatric patients. J Clin Microbiol 42: 5189–5198. 15528714 10.1128/JCM.42.11.5189-5198.2004 PMC525141

[23] Bialasiewicz S, Lambert SB, Whiley DM, Nissen MD, Sloots TP (2009) Merkel cell polyomavirus DNA in respiratory specimens from children and adults. Emerg Infect Dis 15: 492–494. 19239774 10.3201/eid1503.081067 PMC2681122

[24] Khan G, Philip PS, Al Ashari M, Houcinat Y, Daoud S (2011) Localization of Epstein-Barr virus to infiltrating lymphocytes in breast carcinomas and not malignant cells. Exp Mol Pathol 91: 466–470. 21600202 10.1016/j.yexmp.2011.04.018

[25] Dorries K (1998) Molecular biology and pathogenesis of human polyomavirus infections. Dev Biol Stand 94: 71–79. 9776228

[26] Boldorini R, Brustia M, Veggiani C, Barco D, Andorno S, et al (2005) Periodic assessment of urine and serum by cytology and molecular biology as a diagnostic tool for BK virus nephropathy in renal transplant patients. Acta Cytol 49: 235–243. 15966283 10.1159/000326143

[27] Dorries K, Vogel E, Gunther S, Czub S (1994) Infection of human polyomaviruses JC and BK in peripheral blood leukocytes from immunocompetent individuals. Virology 198: 59–70. 8259683 10.1006/viro.1994.1008

[28] Ferrante P, Caldarelli-Stefano R, Omodeo-Zorini E, Vago L, Boldorini R, et al (1995) PCR detection of JC virus DNA in brain tissue from patients with and without progressive multifocal leukoencephalopathy. J Med Virol 47: 219–225. 8551272 10.1002/jmv.1890470306

[29] Hachana M, Amara K, Ziadi S, Gacem RB, Korbi S, et al (2011) Investigation of human JC and BK polyomaviruses in breast carcinomas. Breast Cancer Res Treat 10.1007/s10549-011-1876-5 22108781

[30] Hachana M, Trimeche M, Ziadi S, Amara K, Korbi S (2009) Evidence for a role of the Simian Virus 40 in human breast carcinomas. Breast Cancer Res Treat 113: 43–58. 18205041 10.1007/s10549-008-9901-z

[31] Onorato IM, Morens DM, Martone WJ, Stansfield SK (1985) Epidemiology of cytomegaloviral infections: recommendations for prevention and control. Rev Infect Dis 7: 479–497. 2994198 10.1093/clinids/7.4.479

[32] Cox B, Richardson A, Graham P, Gislefoss RE, Jellum E, et al (2010) Breast cancer, cytomegalovirus and Epstein-Barr virus: a nested case-control study. Br J Cancer 102: 1665–1669. 20407437 10.1038/sj.bjc.6605675 PMC2883146

